# Adequate Wound Care and Use of Bed Nets as Protective Factors against Buruli Ulcer: Results from a Case Control Study in Cameroon

**DOI:** 10.1371/journal.pntd.0001392

**Published:** 2011-11-08

**Authors:** Jordi Landier, Pascal Boisier, Félix Fotso Piam, Blanbin Noumen-Djeunga, Joseph Simé, Fidèle Gaetan Wantong, Laurent Marsollier, Arnaud Fontanet, Sara Eyangoh

**Affiliations:** 1 Service de Mycobactériologie, Centre Pasteur du Cameroun, Réseau International des Instituts Pasteur, Yaoundé, Cameroon; 2 Service d'Epidémiologie et de Santé Publique, Centre Pasteur du Cameroun, Réseau International des Instituts Pasteur, Yaoundé, Cameroon; 3 Unité d'Epidémiologie des Maladies Emergentes, Institut Pasteur, Paris, France; 4 Hôpital de District de Bankim, Bankim, Adamaoua, Cameroon; 5 Groupe d'Etude des Interactions Hôte-Pathogène, Université d'Angers, Angers, France; 6 Conservatoire National des Arts et Métiers, Chaire Santé et Développement, Paris, France; National Institute of Parasitic Diseases China CDC, China

## Abstract

**Background:**

Buruli ulcer is an infectious disease involving the skin, caused by *Mycobacterium ulcerans*. Its exact transmission mechanism remains unknown. Several arguments indicate a possible role for insects in its transmission. A previous case-control study in the Nyong valley region in central Cameroon showed an unexpected association between bed net use and protection against Buruli ulcer. We investigated whether this association persisted in a newly discovered endemic Buruli ulcer focus in Bankim, northwestern Cameroon.

**Methodology/Principal Findings:**

We conducted a case-control study on 77 Buruli ulcer cases and 153 age-, gender- and village-matched controls. Participants were interviewed about their activities and habits. Multivariate conditional logistic regression analysis identified systematic use of a bed net (Odds-Ratio (OR) = 0.4, 95% Confidence Interval [95%CI] = [0.2–0.9], p-value (p) = 0.04), cleansing wounds with soap (OR [95%CI] = 0.1 [0.03–0.3], p<0.0001) and growing cassava (OR [95%CI] = 0.3 [0.2–0.7], p = 0.005) as independent protective factors. Independent risk factors were bathing in the Mbam River (OR [95%CI] = 6.9 [1.4–35], p = 0.02) and reporting scratch lesions after insect bites (OR [95%CI] = 2.7 [1.4–5.4], p = 0.004). The proportion of cases that could be prevented by systematic bed net use was 32%, and by adequate wound care was 34%.

**Conclusions/Significance:**

Our study confirms that two previously identified factors, adequate wound care and bed net use, significantly decreased the risk of Buruli ulcer. These associations withstand generalization to different geographic, climatic and epidemiologic settings. Involvement of insects in the household environment, and the relationship between wound hygiene and *M. ulcerans* infection should now be investigated.

## Author Summary

*Mycobacterium ulcerans* is the causative agent of Buruli ulcer disease, which causes skin wounds and often results in disabilities. The transmission of *M. ulcerans* remains unknown. Environmental and biological studies have gathered evidence that insects could play a role in *M. ulcerans* circulation. A case-control study performed in central Cameroon in 2007 unexpectedly illuminated an association between bed net use and a decreased risk of Buruli ulcer. As this result suggested a potential domestic transmission involving insects, we set up a new study to investigate whether this association existed in Bankim, a newly discovered Buruli ulcer endemic site in northwestern Cameroon. Our results confirm the protective effect of bed nets in this region, despite very different population, environment, and climate factors. They also confirm the role of good hygienic practices, a protective factor repeatedly identified in previous studies. These repeated associations now warrant further research on a possible domestic or peri-domestic transmission of the disease, involving local water collections and possibly insects.

## Introduction

Over the last five years, Buruli ulcer (BU) care has been greatly improved by implementation of antimicrobial therapy and more effective diagnosis methods. However, much about the transmission of this devastating skin disease remains to be learnt. The incidence of BU disease is still on the rise (e.g. in Ghana, Togo and Gabon), and its emergence or increasing incidence have been linked with environmental modifications in rural areas, such as construction of dams or irrigation systems [1]. The main control strategy, based on early detection and early treatment, is hampered in many places by the lack of awareness of the disease and limited access to health services. So far, no primary prevention strategy has been proposed for the population of rural regions where the disease is endemic.

Studies aimed at identifying BU risk factors have repeatedly shown an association between the disease and activities such as wading [2], [3] or washing clothes [4] in marshy areas of stagnant or slow-flowing waters. Farming in short clothes was also found to increase the risk of disease [3], [4]. Using a protected water source was associated with a decreased risk in some settings [2], [5]. Proper care of wounds, such as using alcohol to cleanse wounds, and hygienic practices, such as using soap for bathing, were also repeatedly found protective [2], [3], [6].

In 2006, a case-control study performed in Akonolinga, in the endemic region of the Nyong valley in central Cameroon, showed for the first time that the use of a bed net was associated with protection against BU [3]. This association was strong and independent of the socio-economic level of individuals, as it was found when comparing cases to both village and family controls.

This finding is consistent with a possible role of insects in the transmission of *Mycobacterium ulcerans*. Several water bugs (aquatic Hemipterans) species [7], [8], [9], [10], as well as mosquitoes [11], have been shown to carry *M. ulcerans* in endemic areas. Furthermore, saliva of Hemipterans captured in the environment has been shown to harbor *M. ulcerans*, and the first *M. ulcerans* culture from an environmental sample was obtained from a semi-aquatic Hemipteran [12].

Laboratory experimental infection of mice bitten by infected water bugs provides important biological support for their involvement [9], [13], and mosquitoes-related risk factors have been associated with the disease in Australian epidemiologic studies [14].

Protection against insect bites using a bed net could present a simple, easily implementable means of BU prevention.

We designed this replication study [15] to investigate the association between bed net use and protection against BU in the health district of Bankim, in north-western Cameroon (Figure S1). BU disease was first identified in this region in 2004 [16].

The Bankim BU focus shows several specificities compared to the endemic region of the Nyong river basin in central Cameroon. First, the disease was unknown in the area until recent years (Boussinesq, M. personnal communication). It was reported 15 years after construction of a dam on the Mape River which caused major modifications in the environment and relocations of populations and activities after submersion of villages and farmland. Secondly, the region's geographic, climatic and demographic context differs from that of the forested Nyong River basin. Bankim is located in a transitional zone between forest and savanna and at the foot of the Mambila Mountains; its tropical seasonality is more contrasted than in the equatorial south; and the region is populated by a rich diversity of ethnic groups with specific cultural features. Due to its ecological, demographic and ethnic heterogeneity, Bankim district represented an adequate setting to challenge the hypothesis of bed net use as a protective factor against BU.

Realizing a study in Bankim provided an opportunity to document the other risk factors for BU in this newly discovered focus. It also contributed to raising awareness of BU among Bankim's diverse populations, who considered the disease to result from occult origins and who frequently under-report it.

## Materials and Methods

### Study design and definitions

We conducted a matched case-control study in the district of Bankim, Adamaoua region, Cameroon.

#### Cases

A probable case of BU was defined as a patient presenting with active or inactive BU disease, diagnosed between 1 January 2007 and 15 August 2009 by trained health personnel (specially trained medical doctor and nurse) and treated according to WHO recommendations at the district hospital in Bankim.

A confirmed case was defined as a probable case with laboratory evidence of *M. ulcerans* infection, proved by identification of acid-fast bacilli in stained smears from swabs taken from lesions, amplification of IS*2404* by polymerase chain reaction, or both. Analyses were performed at the Mycobacteria Reference Laboratory in Centre Pasteur du Cameroun, Yaoundé, as described previously [3].

Cases originating from outside the health district were not included in the study. Cases reporting having lived with lesions for 10 years or more were not included, since reporting of exposures before the disease would have been inaccurate and comparison with activities of controls over the last year would have been irrelevant.

#### Controls

Two kinds of controls were enrolled.

An eligible community control was defined as a person without signs or symptoms of active or inactive BU and who was not related to a BU case (sibling, parent or living in the same house). Two controls matching cases for age (+/−2 years), sex, and village were randomly selected. Census data was collected in June 2009 by the district “community health agents” during activities of the National Onchocerciasis Control Program. For each village, a list presenting the name, gender and age of each inhabitant was obtained. Using this list, we assigned a number to each person in the village matching a case for age and gender, and then drew randomly five numbers among all numbers assigned. Controls were contacted in the order they were drawn, received preliminary information from the village “community health agent”, and were given an appointment for an interview. If this person failed to show up at two successive appointments, we visited the next control on the list until two controls were interviewed. In three instances where census data were not available, we selected as controls the first persons matching age and sex criteria and living in a randomly chosen house in the village.

An eligible familial control was defined as a healthy member of the family closest in age to the case, within the same age class (under 5, between 5 and 12, between 13 and 18, over 18). Siblings of the case were preferentially included, but more distantly related people living in the same household could be included in the absence of siblings.

### Ethics statement

Study enrollment was voluntary. Information was provided first to the village population then individually to the participants. Written informed consent was obtained from case patients and control subjects or from their parents or guardians if participants were younger than 18 years. All BU case patients had received or were currently receiving free treatment for BU at the district hospital, a local clinic or the reference hospital for BU in Ayos. The study protocol was approved by the National Ethics Committee and the Cameroon Ministry of Public Health.

### Sample size

The sample size computed for this matched case-control study required 71 groups of one case and two community controls to document an odds-ratio of 0.4 or less as statistically significant, with the following assumptions: proportion using bed nets in the control population = 0.5 (as hypothesized based on previous data available [3]), case/control ratio = 0.5, power (1−β) = 0.8, significance level (alpha) = 0.05, correlation of exposure between groups in the case-control set (phi) 0.2, calculated using PASS (2008, NCSS, trial version). The requested sample size was the same for the case/family control arm, although we anticipated difficulties for including 2 matched controls in some households.

### Data collection

From June 4 to August 16, 2009, study personnel administered a standard questionnaire to participants concerning demographic, environmental and behavioral risk factors. The questionnaire was adapted from Pouillot et al. [3] to fit to the Bankim setting and further questions about bed nets were introduced.

Questions addressed fishing, bathing, washing clothes and domestic water supply activities, as well as farming activities. For all those activities, frequency, localization, and clothes worn were documented, as well as activity-specific details. Frequency was addressed as “daily”, “weekly”, “monthly”, “less than monthly” or “never”. Less than monthly frequencies were rarely reported, thus weekly, monthly, and less than monthly frequencies were grouped as “rare to often”. “Daily” was coded as “always”. Bed net use was considered systematic when people reported sleeping under a bed net every night, all year long. When bed net use was reported seasonal, or irregular, it was classified as “occasional”. All questions were close-ended and asked in French whenever possible. Translations into common local languages (Pidgin-english, Fulfuldé and Tikar) were provided by the same interpreter when needed. Cases were interviewed about their activities during the year before the onset of disease, while controls were asked to report their activity during the previous year.

### Statistical methods

Data were analyzed using STATA version 10.0 (STATA Corp. College Station, Texas). Univariate analysis was performed using conditional logistic regression to calculate odds-ratio (OR) and 95% confidence intervals (95%CI). Missing values for a given variable were not included in the univariate analysis of this variable. Variables with p-values lower than 0.25 in univariate analysis were included in a multivariate model.

Multivariate analysis was performed on these variables using multivariable conditional logistic regression after a first step of intra-exposure category selection. Independently associated variables among an exposure category were in a second step introduced in a multi-exposure category model. At each of the two steps, a stepwise backwards procedure was used to remove from the model the variable with the highest p-value until all p-values were less than 0.05. This procedure (clogit) compared nested conditional logistic regression models by a likelihood ratio test.

In the final model, the “interviewer” effect was assessed by creating a variable “interviewer” coded “0” or “1”, and by testing whether odds ratios of the significant associations of the final model were “modified” when adding an interaction term between each variable of the final model and the variable “interviewer”. Interactions between variables of the final model and age or gender variables were tested using the same method. Age was recoded in 4 categories according to quartiles (<10, 10–13, 14–35, >35).

Preventive fraction was defined as the proportion of cases that could be avoided by systematic exposition of the population to a given protective factor. For instance, the preventive fraction for adequate wound hygiene is the proportion of Buruli ulcer cases that would be avoided if every person in the population cleansed their wounds with soap regularly. Preventive fraction was calculated for each protective factor using the odds ratio estimated in the final model according to the formula developed by Miettinen [17] and Bruzzi [18]:

Preventive Fraction = [P(having disease/unexposed)−P(having disease)]/P(having disease/unexposed) = P(Exposed)×(1−OddsRatio).

## Results

### Participants

**Figure 1:**
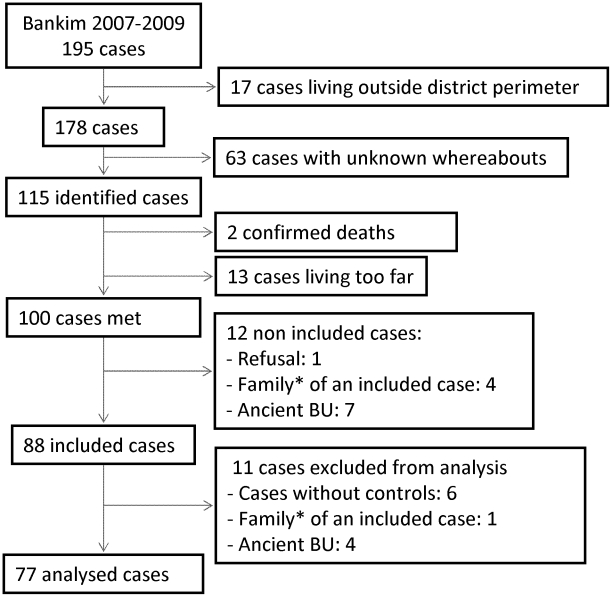
Flow chart of Buruli ulcer cases recruitment. * only one case per family was included in the analysis.

Of the 195 probable Buruli ulcer cases recorded in Bankim district hospital since January 2007, 100 cases were contacted, 88 were questioned, and 77 retained for analysis. Only one BU patient refused to participate. Cases were not included or not considered for analysis if they reported having suffered from BU for ten years or longer (n = 11), if one relative (parent, child or sibling) was already included in the study (n = 5) or if their distant location did not allow us to investigate community controls (n = 6). Details of the inclusion process are provided in Figure 1.

Community controls were randomly selected in the population census for 74 cases. For 3 cases living in villages for which census data were not available, controls were included from randomly selected houses. The main reasons for controls not participating were long-term or repeated absence (n = 30, 45%), refusal (n = 14, 21%), unable to be found (n = 8, 12%), or related to a case (n = 5, 7%).

The main analysis was performed on 77 cases (72 probable and 5 laboratory confirmed cases) matched with 153 community controls. Out of 77 cases analyzed, 76 were matched with two controls and one case was matched with one control only.

Familial controls were recruited when available, i.e. mainly for children. Case-familial control analysis was performed on 37 cases and 49 controls. These were distributed as follows: 12 groups of one case and two controls and 25 groups of one case and one control. Among controls, 42 were siblings, 5 were other relatives (half-brother or sister, cousin) and two were unrelated children living in the same household.

### Characteristics of cases

Most patients were healed, except for 16, who presented with lesions still not scarred (Table S1). Five cases could be confirmed, but laboratory confirmation could not be obtained for 11 who were already undergoing treatment.

About as many cases were men (n = 40) as women (n = 37). Median age of analyzed cases was 14 years (Inter-quartile Range (IQR) = [10–36.5]).

Lesions classically presented on lower (n = 39, 49%), and upper limbs (n = 29, 38%). Only 3 patients presented with multiple lesions, and 8 (10%) presented with head or trunk lesions. Median (IQR) time from onset of disease to day of interview was 17 (9–26) months, ranging from 15 days to 7 years.

The majority of patients did not associate their disease with any particular circumstance, but 9 linked it to a wound and 17 to insect bites.

The overall population of the study, presented in Table 1, showed an important heterogeneity of ethnic groups and origins. Tikari and Kwadja farmers are indigenous and represented 33% of controls, but Yamba and Mambila farmers originating from neighboring regions were also well represented, respectively 29% and 12% of the control population, respectively. Kotoko people, originating from the Extreme-North Cameroon, are also found in Bankim, where they settled on the banks of the Mappé Lake and currently handle the commercial fishing business. Fulani-related Mbororos live usually in settlements in the savanna where they traditionally raise cattle. Some of them practice transhumant pastoralism.

**Table 1:** Univariate analysis of selected demographic variables for Buruli ulcer in Bankim, Community-matched case-control study.

Variables		Cases		Controls		OR*	[95%CI]*	p*
		N	(%)	N	(%)			
**Gender**	Men	40	(52)	79	(52)	Matching variable		
	Women	37	(48)	74	(48)			
**Age (years)**	median [IQR]	14 [10–34]		13 [9–35]		Matching variable		
**Ethnic group**	Tikari/Kwadja	26	(34)	51	(33)	1	Reference	0.52
	Fulani (Mbororo/Fulbe)	2	(3)	9	(6)	0.4	[0.1–2.0]	
	Mambila	7	(9)	19	(12)	0.7	[0.2–2.1]	
	One Tikari parent	6	(8)	9	(6)	1.4	[0.5–4.2]	
	Yamba	26	(34)	44	(29)	1.3	[0.5–3.0]	
	Kotoko	3	(4)	11	(7)	0.4	[0.1–2.0]	
	Other	7	(9)	10	(7)	1.6	[0.5–4.6]	
**Main activity**	Activity in the bush	29	(38)	62	(41)	1	Reference	0.32
	School	35	(45)	72	(47)	1.1	[0.4–3.6]	
	Activity in the town	7	(9)	10	(7)	1.4	[0.5–4.0]	
	Child <5 years	6	(8)	9	(6)	NA	NA	
**Fishing activity**	Y/N	27	(35)	64	(42)	0.7	[0.4–1.3]	0.24
**Farming activity**	Y/N	63	(82)	131	(86)	0.7	[0.3–1.6]	0.35
**Education level**	None	18	(23)	33	(22)	1	Reference	0.70
	Primary (or coranic)	19	(25)	47	(31)	0.7	[0.3–1.9]	
	Secondary	9	(12)	12	(9)	1.2	[0.4–4.4]	
	Children <12 years	31	(40)	61	(40)	NA	NA	
**BCG scar**	Present Y/N	29	(38)	76	(50)	0.6	[0.3–1.1]	0.08
**Compound environment**								
**-Coffee trees**	Y/N	4	(5)	18	(12)	0.4	[0.1–1.2]	0.09
**-Water collection**	Y/N	17	(22)	16	(10)	2.6	[1.1–5.8]	0.02
**-Domestic animals**	Y/N	61	(79)	105	(69)	2.6	[1.2–5.7]	0.01
**No data**		1		0		–	–	–

*Odds-Ratio, 95% confidence interval and p-value obtained using conditional logistic regression.

Adults principally engaged in farming activities. Most children between 6 and 12 years old reported going to school (94%), and a majority of them participated in farming during weekends and holidays or during seasons when additional workforce was required (85%). Education level was low with few persons over 12 years of age having secondary education.

### Community-matched case-control study

#### Univariate analysis

Neither ethnic group, activity nor education level was significantly associated with BU (Table 1). A history of BCG vaccination, assessed through the presence of a scar on the forearm, showed a nearly significant protection against BU. Compound environment was associated with an increased risk when small domestic animals such as poultry, ducks, goats or pigs, were present (Odds Ratio (OR) 95% Confidence Interval [95%CI] = 2.6 [1.2–5.7], p = 0.01) and when the compound was close to a water body (OR [95%CI] = 2.6 [1.1–5.8], p = 0.02). Use of specific light source (petrol lamp, electricity) and whether it burnt overnight or not, were not associated with the disease.

Exposure to insects was assessed by questioning about specific insects bites (Table 2). Cases reported more frequently being bitten by hematophagous insects belonging to the *Chrysops* genus (Tabanidae), but the association was not significant. BU was significantly associated with reporting history of small skin lesions due to scratching after an insect bite (OR [95%CI] = 2.1 [1.2–3.7], p = 0.01). Mosquito coils were seldom used and no association with BU was found.

**Table 2:** Univariate analysis of selected individual variables for Buruli ulcer in Bankim, Community-matched case-control study.

Variables		Cases		Controls		OR*	[95% CI]*	p*
		N	(%)	N	(%)			
**Insect bites**								
**- Chrysops sp.**	Never	7	(9)	24	(16)	1	Reference	0.22
	Rare to often	51	(66)	100	(65)	1.8	[0.7–4.5]	
	Every day	17	(22)	25	(16)	2.6	[0.9–7.6]	
	Doesn't know	2	(3)	4	(3)	–	–	
**- Simulium sp.**	Y/N	63	(82)	120	(78)	1.4	[0.7–2.8]	0.40
**- cause scratch wounds**	Y/N	49	(64)	70	(46)	2.1	[1.2–3.7]	0.01
**Protection**								
**- Mosquito coil use**	Never	58	(75)	122	(80)	1	Reference	0.49
	Rare to often	15	(19)	21	(14)	1.5	[0.7–3.3]	
	Every day	4	(5)	10	(7)	0.9	[0.3–2.9]	
**- Bed net use**	Never	32	(42)	59	(39)	1	Reference	0.01
	Rare to often	15	(20)	11	(7)	2.5	[1.0–6.4]	
	Every day	30	(39)	81	(53)	0.6	[0.3–1.2]	
	ND	0		2		–	–	
**Bed net characteristics**								
**- Insecticide treated**	No net	32	(42)	59	(39)	1	Reference	0.74
	Never	28	(36)	66	(43)	0.8	[0.4–1.6]	
	Not recently	6	(8)	9	(6)	1.3	[0.4–3.9]	
	Recently	11	(14)	18	(12)	1.2	[0.4–3.1]	
	ND	0		1		–	–	
**- With holes**	No net	32	(92)	59	(39)	1	Reference	0.66
	Yes	27	(35)	62	(41)	1.1	[0.5–2.4]	
	No	18	(23)	31	(20)	0.8	[0.4–1.6]	
	ND	0		1		–	–	
**Wound treatment practices**								
**- use leaves**	Yes/No	34	(44)	76	(50)	0.7	[0.4–1.4]	0.36
**- use soap**	Yes/No	11	(14)	58	(38)	0.1	[0.1–0.4]	<0.001
**- use alcohol**	Yes/No	20	(26)	63	(41)	0.5	[0.3–0.9]	0.02
**- use ointment**	Yes/No	42	(55)	77	(50)	1.1	[0.6–2.0]	0.67
**- no treatment**	Yes/No	12	(16)	14	(9)	1.9	[0.8–4.4]	0.14
**Treatment frequency**	No treatment	8	(10)	11	(7)	1	Ref	0.70
	≤1 time/week	20	(26)	41	(27)	0.7	[0.2–1.9]	
	>1 time/week	49	(64)	99	(65)	0.6	[0.2–1.8]	
	ND	0		2		–	–	
**Dressing frequency**	No bandage	39	(51)	58	(38)	1	Ref	0.03
	≤1 time/week	14	(18)	22	(14)	1.0	[0.4–2.3]	
	>1 time/week	22	(29)	73	(48)	0.5	[0.2–0.9]	
	ND	2		0		–	–	
**Take tablets**	Yes/No	19	(25)	49	(32)	0.6	[0.3–1.2]	0.16

*Odds-Ratio, 95% confidence interval and p-value obtained using conditional logistic regression.

Selected variables are presented among the following categories: insect exposure, insect protection and wound care.

When compared to never using a bed net, systematic bed net use was associated with protection from BU and occasional use was associated with an increased risk (systematic/none: OR [95%CI] = 0.6 [0.3–1.2], occasional/none: OR [95%CI] = 2.5 [1.0–6.4]; p = 0.01). Most bed nets had been bought from the local market and were not insecticide-treated. Net insecticide treatment did not appear associated with better protection, nor did the absence of holes.

Exposure to water was assessed for various activities (Table 3). Fishing was a common secondary occupation among all categories of population but the study included few professional fishermen. Fishing activity, season, place or techniques used were not found to have statistically significant association with the disease.

**Table 3:** Univariate analysis of selected water exposures for Buruli ulcer in Bankim, Community-matched case-control study.

Activities		Cases		Controls		OR*	[95%CI]*	P*
		N	(%)	N	(%)			
**Fish**	Never	40	(52)	75	(49)	1	Reference	0.84
	Rare to often	17	(22)	38	(25)	0.8	[0.4–1.7]	
	Every day	20	(26)	40	(26)	0.9	[0.4–1.9]	
**Bathe in water collections**								
**-for hygiene**	Never	23	(30)	58	(38)	1	Reference	0.45
	Rare to often	15	(19)	26	(17)	1.5	[0.6–3.5]	
	Every day	39	(51)	69	(45)	1.5	[0.8–3.0]	
**Bathing place**	Barrage Y/N	13	(17)	24	(16)	1.1	[0.5–2.5]	0.78
	Mbam Y/N	8	(10)	5	(3)	4.4	[1.2–17]	0.02
	Other river Y/N	34	(44)	68	(44)	1	[0.5–1.8]	1
**-for leisure**	Never	35	(45)	83	(54)	1	Reference	0.28
	Rare to often	16	(21)	23	(15)	1.9	[0.8–4.4]	
	Every day	26	(34)	43	(28)	1.5	[0.7–3.3]	
	ND	0		4	(3)	–		
**Wash clothes**	Never	6	(8)	20	(13)	0.6	[0.2–1.8]	0.16
	Rare to often	15	(20)	18	(12)	1.6	[0.8–4.2]	
	Every day	56	(73)	115	(75)	1	Reference	
**Carry water for household**	Never	17	(22)	26	17	1.6	[0.6–4.2]	0.49
	Rare to often	19	(25)	44	(29)	0.9	[0.4–1.9]	
	Every day	41	(53)	83	(54)	1	Reference	
**Domestic water**	Unprotected	65	(84)	116	(76)	1	Reference	0.23
**source**	Protected	6	(8)	17	(11)	0.5	[0.2–1.7]	
	Not applicable	6	(8)	20	(13)	0.5	[0.2–1.4]	
**Clothes worn during household water activities**								
**Lower body**	Long	40	(52)	78	(51)	1	Reference	0.36
	Short or none	32	(42)	59	(39)	1.0	[0.5–2.0]	
	Not applicable	4	(5)	15	(10)	0.4	[0.1–1.6]	
	ND	1		1		–	–	
**Upper body**	Long	9	(12)	15	(10)	1	Reference	0.34
	Short or none	63	(82)	122	(80)	1.2	[0.5–3.0]	
	Not applicable	4	(5)	15	(10)	0.4	[0.1–1.6]	
	ND	1		1		–	–	
**Shoes**	No	14	(18)	7	(5)	6.7	[1.8–24.3]	0.002
	Yes	58	(75)	128	(84)	1	Reference	
	Not applicable	4	(5)	15	(10)	0.7	[0.2–2.9]	
	ND	1		3		–	–	

*Odds-Ratio, 95% confidence interval and p-value obtained using conditional logistic regression.

Bathing activities were separated into bathing for hygiene and for leisure. Bathing for hygiene was statistically associated with an increased risk of BU when baths were taken in the Mbam, a river flowing south from the district (OR [95%CI] = 4.4 [1.2–17], p = 0.02). Bathing in the barrage lake or other water bodies was not associated with a statistically significant change in the risk of disease. Bathing for leisure was a common activity for nearly all children and did not significantly increase risk.

Domestic water supply and clothes washing activities were investigated, but no significant difference in exposure was found between cases and controls. Water sources were mainly rivers, streams or wells. Protected water sources were limited to urban compounds and not necessarily used for all activities. Whether unprotected water sources were running or stagnant was difficult to assess, but no statistically significant association was found between BU and declared characteristics of the water source.

Not wearing shoes during domestic water supply or clothes washing was significantly associated with an increased risk of BU (OR [95%CI] = 6.7 [1.8–24.3], p = 0.002). Type of clothing or shoes worn during any other water-related activity was not associated with a significant risk of BU.

Almost all cases and controls participated in farming activities (Table 4). Youngest children were considered participating when they accompanied their parents. Nearly all farmers planted corn. Growing other food crops was associated with a decreased risk of disease: growing cassava (OR [95%CI] = 0.4 [0.2–0.7], p = 0.001) or beans (OR [95%CI] = 0.5 [0.3–1.0], p = 0.05) presented a significantly decreased risk. Growing banana (sweet or plantain), groundnut and tubers like yam, sweet potato or taro was also associated with a small protection that did not reach significance level. In contrast, commercial crops like coffee and pepper were not associated with disease.

**Table 4:** Univariate analysis of farming activity variables for Buruli ulcer in Bankim, Community-matched case-control study.

		Cases		Controls		OR*	[95%CI]*	p*
		N	(%)	N	(%)			
**Farming activities** +	Y/N	71	(92)	145	(95)	0.6	[0.2–2.0]	0.4
**Cultures**	Corn Y/N	71	(92)	143	(93)	0.8	[0.3–2.4]	0.71
	Coffee Y/N	20	(26)	47	(31)	0.8	[0.4–1.5]	0.44
	Banana Y/N	21	(27)	55	(36)	0.7	[0.4–1.2]	0.19
	Cassava Y/N	45	(58)	122	(80)	0.4	[0.2–0.7]	0.001
	Ground-nut Y/N	56	(73)	124	(81)	0.6	[0.3–1.2]	0.12
	Beans Y/N	14	(18)	46	(30)	0.5	[0.3–1.0]	0.05
	Tubers Y/N	33	(43)	80	(52)	0.7	[0.4–1.2]	0.15
	Pepper Y/N	12	(16)	33	(22)	0.7	[0.3–1.4]	0.27
**Farming area**	Mbam Y/N	44	(57)	82	(54)	1.3	[0.6–2.8]	0.52
	Barrage Y/N	22	(29)	54	(35)	0.6	[0.3–1.3]	0.19
**Walking time to field**	<45 min	22	(29)	67	(44)	1	Reference	0.10
**(self estimated)**	Around 1 h	22	(29)	34	(22)	1.9	[0.9–3.9]	
	1.5 h and more	25	(32)	40	(26)	2.0	[0.9–4.4]	
	Unknown	6	(8)	8	(5)	–	–	
	Not going to field	2	(3)	4	(3)	–	–	
**Submersed field**	Yes/No	20	(26)	27	(18)	1.7	[0.9–3.4]	0.12
**Sleep at the field**	Yes/No	21	(27)	22	(14)	2.4	[1.1–5.0]	0.02
	ND	2		8		–	–	
**Own a “garden”**								
**Nearby compound**	No garden	55	(71)	120	(78)	1	Reference	0.10
	No	13	(17)	26	(17)	1.2	[0.5–2.8]	
	Yes	9	(12)	6	(4)	3.4	[1.1–10.6]	
	ND	0		1		–	–	
**Water garden**	Yes/No	19	(25)	19	(12)	2.8	[1.2–6.4]	0.01
	ND	0		1		–	–	

**+:** children were considered to engage in farming activities when they accompanied their parents to the fields.

*Odds-Ratio, 95% confidence interval and p-value obtained using conditional logistic regression.

Cases were more likely than controls to have a longer walking time to their fields and to sleep there. They more frequently reported cultivating areas that flooded during the rainy season. Cases also more often declared having a garden or a vegetable patch near their houses, and watering these plots was associated with a significantly increased risk.

Clothing worn during farming activities was not associated with a risk of BU.

Wounds were frequent in the population, and were usually acquired during farming activities (Table 3). Controls reported being wounded while clearing the field more often than cases.

Several wound treatments were associated with a change in BU risk: using alcohol or soap to cleanse the wound were protective practices, while using no treatment led to an increased risk of disease. Rubbing leaves (usually the sap of a common Lamiaceae referred to as “benjamin”) or applying ground tablets or ointment bought on the market was not significantly associated with the risk of BU disease. Dressing the wound was a protective practice when the bandage - either a piece of cloth or adhesive bandage - was regularly changed.

#### Multivariate analysis

For multivariate analysis, the main exposure “bed net use” was recoded in two categories, “Systematic bed net use Yes/No” by pooling the two categories “Never” and “Rare to often”, which were both associated with an increased risk of disease in the univariate analysis (Table 5).

**Table 5:** Multivariable model for risk factors for Buruli ulcer in Bankim, Cameroon, 2007–2009, Community-matched case-control study.

		AdjOR	95%CI	p
**Bathing for hygiene in Mbam river**	Yes/No	6.9	[1.4–34.7]	0.02
**Cleanse wounds with soap**	Yes/No	0.1	[0.03–0.3]	<0.001
**Grow cassava**	Yes/No	0.3	[0.2–0.7]	0.005
**Always sleep under a bed net**	Yes/No	0.4	[0.2–0.9]	0.04
**Report scratching wounds after insect bites**	Yes/No	2.7	[1.4–5.4]	0.004

Log likelihood ratio: 59.32; Likelihood ratio test: LR chi-square = 48.11, p<10^−4^.

Independent factors associated with an increased risk of BU were bathing for hygiene in the Mbam River (OR [95%CI] = 6.9 [1.4–35], p = 0.02) and reporting small scratch wounds after insect bites (OR [95%CI] = 3.8[1.0–14], p = 0.03). Factors independently associated with a decreased risk were systematic use of a bed net (OR [95%CI] = 0.4 [0.2–0.9], p = 0.04), use of soap to cleanse wounds (OR [95%CI] = 0.1 [0.03–0.3], p<0.001) and growing cassava (OR [95%CI] = 0.3 [0.2–0.7], p = 0.005).

No significant interaction was found in the final model between variables and a variable “interviewer”. No significant interaction was found between these variables and age category or gender. Detailed results of variable selection process are provided in supplementary text S1.

### Family-matched case-control study

Univariate analysis showed a few associations that did not reach significance level (table S2). Of note among those were: bed net use which was associated with a decreased risk (OR [95%CI] = 0.4 [0.1–1.4], p = 0.13); watering a garden, associated with an increased risk (OR [95%CI] = 3.1 [0.6–15], p = 0.13); and reporting scratch wounds after insect bites, associated with an increased risk of BU (OR [95%CI] = 2.3 [0.8–6.7], p = 0.10). No significant association with BU was revealed in multivariate analysis (table S2).

### Preventive fraction

If we assume that the relationship between a protective factor and risk decrease is truly causal, we can calculate the proportion of cases in the general population (represented by the controls) prevented by this protective factor, based on the relative risk and the frequency of the factor in the general population (again, represented by controls).

The preventive fraction for systematic bed net use was 32% (proportion of controls systematically using a bed net: 53%, OR = 0.4). Likewise, the fraction of cases prevented by adequate wound hygiene practices (using soap to cleanse wounds) was 34% (proportion of controls using soap: 38%, OR = 0.1).

## Discussion

This study identified practices that demonstrate statistically significant associations with Buruli ulcer in a newly endemic area of northwestern Cameroon. Systematic bed net use, adequate wound hygiene practices, and growing cassava were associated with a decreased risk of disease. Bathing for hygiene in the Mbam River was associated with an increased risk, as well as reporting frequent occurrence of scratch wounds following insect bites.

### Validity of the study

The chosen setting, Bankim district in the Adamaoua region, contrasts with the previous study site. Akonolinga, the previous study site, was characterized by a relative homogeneity: 80% of the population belonged to the same ethnic group, the region was largely forested and water-related activities were centered on a major river, the Nyong [3]. By contrast, Bankim was home to numerous ethnic groups with wider variation in environmental, cultural and health practices. Bankim's environment is a highly varied mosaic of savanna, mountain, and forests. Water-related activities took place in the Mappé reservoir, the Mbam River and many other smaller water bodies (Figure S1).

Our rigorous sampling method, which used census data and repeated appointments with controls, allowed us to capture accurately the heterogeneities of populations and activities in Bankim and to minimize control recruitment associated biases [19]. Case recruitment was performed within the entire district and was not restricted to the most accessible areas.

Similar to case-control studies on BU, the present study has several limits inherent in its methodology and context. First, even if we are confident that our sampling method prevented most sampling biases, memory bias could have occurred: cases were interviewed on their activities during the period preceding the onset of disease and could therefore remember them differently from controls, who were interviewed on their activities over the previous year. However, we included mainly cases with disease diagnoses during the two previous years. This ensured that cases and controls would recall their activities on similar time scales and minimized differential bias. Furthermore, having suffered Buruli ulcer disease could have influenced cases' answers to questions, such as the occurrence of scratch wound as discussed below (rumination bias, [19]). However, most of our identified risk or protective factors are related to less subjective behavioral parameters, which are less likely to be influenced by the disease status.

Finally, this study was performed in a region that is distant from national or regional level healthcare facilities, and where the disease was only recently reported. But in 2007, because of the increasing number of cases, Bankim health personnel were trained at the national Buruli ulcer reference hospital in Ayos on case detection, systematic recording and basic management of cases according to WHO guidelines. These trained personnel implemented these practices in the district. During the period of the present study, a systematic procedure for laboratory case confirmation was set up although only a few cases were confirmed microbiologically. However, clinical diagnosis has been demonstrated to be highly specific when performed by trained health personnel, so that we are confident that almost all clinically-diagnosed Buruli cases were true cases [20]. In a previous study in Akonolinga, Cameroon, analysis of probable and confirmed cases yielded the same risk factors as confirmed cases only [3].

### Protective factors for Buruli ulcer in Bankim

#### Protection using a bed net

This study confirms the association between bed net use and protection against BU, previously shown in Akonolinga, in central Cameroon [3].

The previous study in Akonolinga had shown that the strong association between use of a bed net and protection against BU (OR [95%CI] = 0.4 [0.2–0.8] persisted between cases and controls within the same household (OR [95%CI] = 0.1 [0.3−0.03]), suggesting that it was not confounded by socio-economic status. A larger proportion of adults was included in the present study which made it more difficult to recruit siblings as familial controls. Statistical power is consequently low in the family-matched case-control study. However, use of a bed net still presents a protective effect in this sub-study, which, although not significant, has the same magnitude (OR [95%CI] = 0.4 [0.1–1.4]) as in the community-matched study (OR [95%CI] = 0.4 [0.2–0.9]). While socio-economical status might have confounded the association in the community-matched study, if for instance, participants using bed nets were less likely to have activities exposing them to *M. ulcerans*, it is unlikely to be the case in the family-matched study. Indeed, in this study, cases and their familial controls share the same socio-economical level.

Here we demonstrate that the association between bed net use and protection against BU withstands generalization in a different climatic, geographic, environmental, and ethnic context. This replicative study greatly increases the positive predictive value of the association between bed net use and decreased risk of BU [15].

Few epidemiological studies have investigated the possible role of a bed net as a protective factor against BU in Africa. In Ghana, no significant association was found between bed net use and protection against BU, but the proportion of bed net users in the general population was low (25%), and the 116 pairs of cases and controls only yielded a power 67% to show an OR of 0.4 or less [6]. Furthermore, categories of bed net use grouped “sometimes” and “always” together, probably because of overall infrequent use. In our study, always using a bed net was protective, while sometimes using one was not. Out of two risk-factor studies in Benin, one did not address the question of bed net use [5], while the other showed a small risk increase for bed net users in univariate analysis which disappeared when adjusting for other variables in a multivariate model [2].

The preventive fraction for systematic bed net use was 32% in this study, and reached 43% in the previous study performed in Akonolinga [3]. If this association proves to be causal, bed net generalization could represent a significant mean of protection against the disease that could lead up to one third reduction in the number of cases.

The protective effect of bed nets could result from two different mechanisms. First, using a bed net could protect from direct inoculation of *M. ulcerans* by an insect vector. This hypothesis is consistent with several results from environmental studies. In Australia, *M. ulcerans* infection is now considered a zoonosis transmitted from possum to human by mosquitoes [21], [22].

Secondly, bed nets could provide a protection by preventing unspecific insect bites. These bites can lead to small lesions after being scratched which would enable *M. ulcerans* to enter the skin when in contact with contaminated environment. Occurrence of scratch wounds following insect bites was indeed associated with an increased risk of disease in our study. This factor was statistically independent of bed net use as shown by the persistence of both variables in the final model. This result suggests that both risk factors may act independently, some insect bites being not preventable by bed nets, for instance those occurring at day time.

The association between bed net use and protection against BU underlines the need to broaden environmental studies to aerial insects and to the domestic environment. It would be relevant to assess the presence of *M. ulcerans* in *Aedes* and *Anopheles* mosquitoes which are important in Africa. Studies on distribution, abundance and *M. ulcerans* positivity of water bugs should not be restricted to the aquatic environment and seek these insects in the domestic environment.

#### Protection from adequate wound hygiene

The strongest protective association was linked to using soap to cleanse wounds. This variable summed up others that implied practices of proper wound care: use of alcohol and frequent bandage changing were also associated with protection, and usually concerned the same people. This association of a decreased risk of Buruli ulcer and good hygiene practices has been found in Ghana [6], Benin [2] and Cameroon [3], indicating that such practices might be one of the most powerful way of avoiding this disease.

This also indicates that while some insects may transmit *M. ulcerans*, other transmission mechanisms are likely to coexist that involve wounds as a port of entry, such as contamination of open wounds by water, or direct inoculation of *M. ulcerans* by a wounding object.

Several mechanisms could explain the decreased risk of disease associated with wound hygiene. Clean wounds heal faster and could be less likely to become a port of entry for the microbe. Frequent wound cleansing could also prevent the survival of the microbe in the skin: since the initial mycobacterial inoculum is expected to be low (3 months median incubation time, [23]) and since the growth rate is very slow, repeated disinfection of skin lesions, or frequent bodily cleansing could help to prevent microbe colonization and subsequent infection, independently of the actual transmission route.

Onset of Buruli ulcer disease after sustaining a wound was reported by 9 cases out of 77 analyzed in this study. This result may well be subject to memory bias. Even so, the role of wounds in the transmission of *M. ulcerans* infection has received little, if any, attention. Symmetrically, the potential role of good hygienic practices has remained poorly investigated by the research community and remains absent from official recommendations, including the WHO BU factsheet [24]. Hygiene-related protective factors have been documented repeatedly and are thus highly relevant to public health. Using published data, we have estimated the preventive fraction for the use of toilet soap for bathing in previous studies and found was 41% in Ghana [6] and 65% in Benin [2]. The preventive fraction for using soap, antibiotic powder or health center care to treat injuries was 53% in Benin [2], while the preventive fraction for using alcohol to treat wounds was 26% in Cameroon [3]. In this study, the preventive fraction of soap use for cleansing wounds was 34%.

While the role of insects in BU transmission remains the cause of much debate and methodological interrogations [25], clarifying the role of hygiene does not seem to have elicited much attention even though most studies confirm its importance. Good hygienic practices could potentially have a major role in community prevention programs.

#### Protection from growing cassava

Our final model also contains an unanticipated protective association with growing cassava. It had also been found in univariate analysis in Ghana [6]. The link between cassava and *M. ulcerans* infection is not obvious; we therefore suspect that in both cases this factor could be associated with an environmental parameter that was not captured by our questionnaires. The main food crop in Bankim is maize, which is highly water-demanding, and Bankim farmers report that cassava grows better in dry, higher grounds. We therefore hypothesize that people growing cassava may work in less swampy, thus less risky areas. Furthermore, bitter cassava is favoured by farmers because insects are less likely to attack it than sweet cassava. Bitter cassava contains toxic cyanoside compounds, and its preparation requires that the tubers be soaked in water, ground to flour and then sundried for several days. During this preparation process, the soaking and drying produce a very intense smell which may possibly repel insects from the domestic area where the preparation takes place.

Of note are also the several univariate associations found between growing certain food crops and a decreased risk of disease. These associations suggest that nutritional status may possibly increase a person's ability to resist *M. ulcerans* infection.

Because this study is the first to show that this factor is significantly associated with a decreased risk of Buruli ulcer disease, we consider that further investigations on the agricultural and nutritional practices are needed before drawing conclusions about its possible role in prevention.

### Risk factors for Buruli ulcer

Bathing for hygiene in the Mbam river was associated with an increased risk of disease. These baths involved people spending long amounts of time near the river for their farming activities and eventually dwelling there during the rainy cultivation season where the waters are high and the banks swampy. Risk increase did not concern leisure baths in the same river, which only took place in the shallow waters during the dry season. This finding is in accordance with numerous previous results identifying slow flowing rivers and stagnant waters as an important source of contamination. The identification of the Mbam river as a risk factor rather than the Mappé reservoir seems at first counter-intuitive, but the reservoir is probably not the direct source of every contamination: some patients had no history of contact with the Mappé lake waters and highest prevalences of BU were not recorded in the fishing settlements on the lake shore but in the villages located between the lake and the Mbam river (figure S1).

Of note, *M. ulcerans* DNA positive water bugs were captured in domestic water collections as well as in the Mappé lake in 2008 [16].

The importance of the peri-domestic water collections as a source of *M. ulcerans* infections remains to be evaluated in terms of *M. ulcerans* presence throughout the year as well as potential vectors or reservoirs breeding sites. Captures targeting water bugs indicated important seasonal variations of abundance and positivity [8], which warrants regular follow-up of microbe and insect populations.

The dam construction may have contributed to BU expansion by providing a broader habitat suitable for proliferation, dissemination and/or survival of *M. ulcerans*, one of its hosts, or an eventual vector. The dam construction in this region impacted drastically on endemicity of onchocerciasis and loasis [26] as well as schistosomiasis (Mouchet F, personal communication).

Increase in BU disease may also result from increased exposure to the environment surrounding the Mbam River where relocated villagers exploit new fields after the dam construction.

The second risk factor identified in this study is more difficult to interpret. Reporting occurrence of scratch wounds after being bitten by an insect was found associated with Buruli ulcer disease. This could be seen as an indication that insect bites are involved in the transmission, either through an insect vector or through unspecific skin lesions from scratching representing a port of entry for *M. ulcerans*. We would however recommend considering this factor with great caution, as it was only reported and actual sensitivity to insect bites was not assessed. Furthermore, BU cases often reported having suffered intense itching before the onset of the lesion, which they often attributed to an insect bite. This event may have led cases to report being more sensitive to insect bites than controls.

Finally, several risk factors commonly evoked for BU did not present statistically significant associations with the disease in this study. Statistical power was probably insufficient to investigate properly several associations, such as education level (very low) and short clothing related associations (infrequently used for farm work or fishing). Bankim environment's heterogeneity probably prevented us from identifying major water sources of exposure: there is a large number of water bodies, and people change both water-related activities and locations throughout the year because these water sources dry up during the dry season. This may explain why in this study, four behavioral factors and only one location-dependent factor had statistically significant associations with BU in the final model.

### Conclusion

Through this study, we have demonstrated that the association between bed net use and protection against BU, found in a previous case-control study in Akonolinga, can be generalized to another climatic, geographical, environmental and ethnic context. We also identified good wound hygiene as a key protective factor, as reported in several previous studies.

These findings support the hypothesis of a domestic or peri-domestic transmission of *M. ulcerans*, by direct (skin lesions) or indirect (insect bites) contamination involving peri-domestic water collections. We believe that it is now of critical interest to confirm or to rule out the domestic vector hypothesis by investigating insects in the domestic and peri-domestic environment of BU endemic foci. Such study would also help to determine if implementation of bed net protection would help prevent BU transmission.

Furthermore, good hygiene appears again as a key protective factor: using soap to cleanse wounds was found protective, while hygiene baths in the Mbam River increased the risk of disease. Hygienic water sources and behaviors appear to be connected. The link between hygiene and *M. ulcerans* transmission merits investigation in greater detail, so as to promote specific hygienic practices through prevention strategies.

Finally, we believe that while clinical research is making progress in developing oral treatment of Buruli ulcer [27], the research on ecology and transmission of *M. ulcerans* infection should be encouraged so that meaningful and effective prevention strategies can be identified and proposed.

## Supporting Information

Figure S1 Map of the Bankim area presenting the number of cases per village from January 2007 to August 2009.
(DOC)

Table S1 Characteristics of the 77 analyzed cases.
(DOC)

Table S2 Case-family matched control study analysis. Univariate and multivariate analysis results of risk factor analysis in Bankim, Cameroon, 2007–2009.
(DOC)

Text S1 Details on multivariate analysis (multivariable conditional logistic regression) for case-community matched control analysis of risk factors for Buruli ulcer in Bankim, Cameroon, 2007–2009.
(DOC)

Protocol S1 Trial protocol.
(DOC)

Checklist S1 CONSORT checklist.
(DOC)
