# Supplementary material for: Adequate Wound Care and Use of Bed Nets as Protective Factors against Buruli Ulcer: Results from a Case Control Study in Cameroon
Source: PLoS Negl Trop Dis. 2011 Nov 8;5(11):e1392. doi: 10.1371/journal.pntd.0001392 (PMC3210760; doi:10.1371/journal.pntd.0001392)
Supplement: Figure S1 — Map of the Bankim area presenting the number of cases per village from January 2007 to August 2009. (DOC) [file pntd.0001392.s001.doc]

**Supplementary figure S1:**


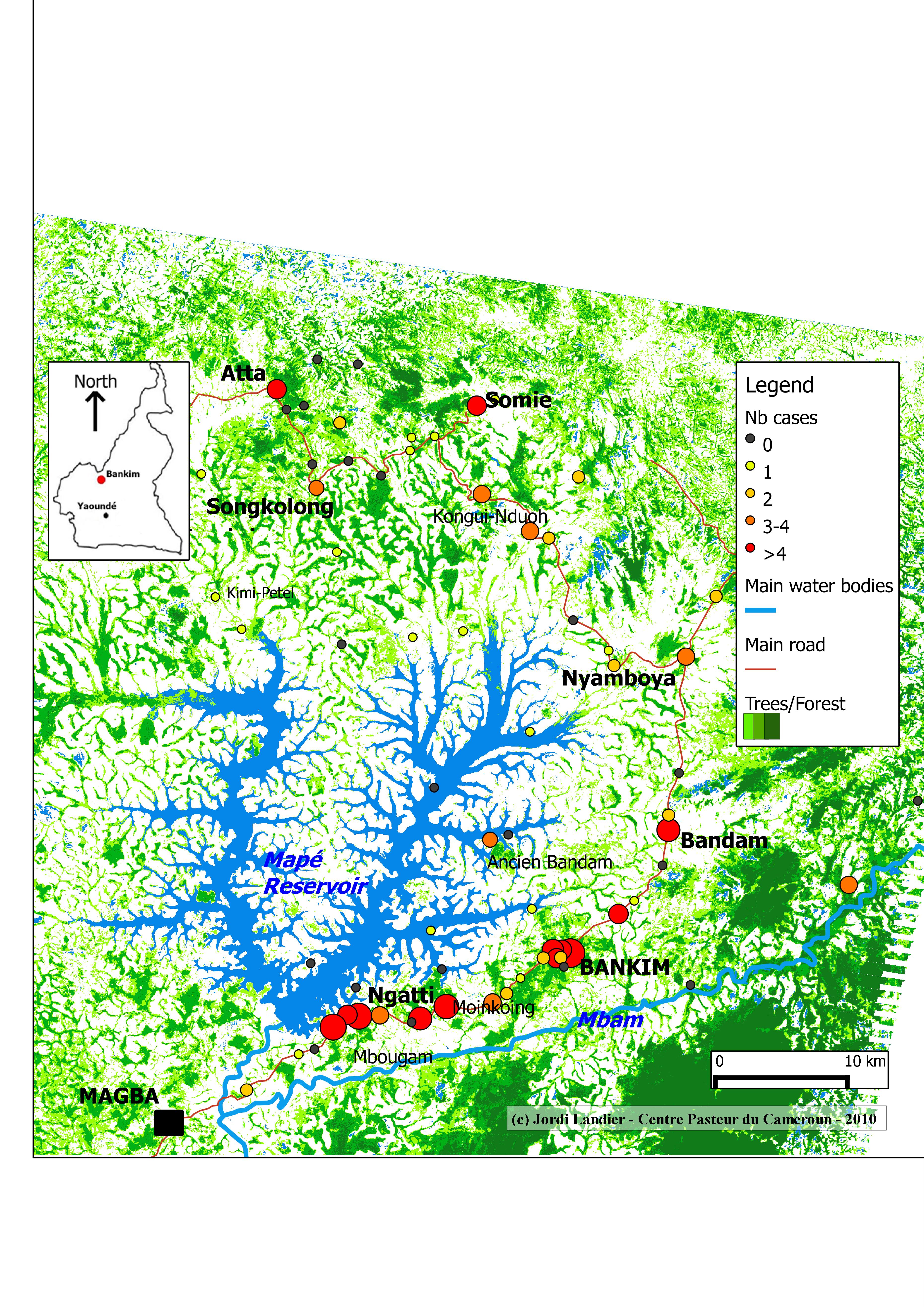


**Figure S1: Map of the Bankim area presenting the number of cases per village from January 2007 to August 2009** (dots proportional to the number of cases).

The map was realized using Quantum-GIS version 1.4. Locations of villages were recorded during the field study using a Garmin 6 GPS. Case numbers were obtained from the Bankim Hospital records. Approximate water and forest areas were isolated using an unsupervised classification algorithm (Multispec, version 3.1, Purdue research foundation) on a Landsat 7 multispectral image from 05 January 2007 (obtained from USGS website).
