# Supplementary material for: Adequate Wound Care and Use of Bed Nets as Protective Factors against Buruli Ulcer: Results from a Case Control Study in Cameroon
Source: PLoS Negl Trop Dis. 2011 Nov 8;5(11):e1392. doi: 10.1371/journal.pntd.0001392 (PMC3210760; doi:10.1371/journal.pntd.0001392)
Supplement: Table S1 — Characteristics of the 77 analyzed cases. (DOC) [file pntd.0001392.s002.doc]

**T**able S1: Characteristics of the 77 analyzed cases

|  |  | N | (%) |
| --- | --- | --- | --- |
| Gender | Male | 40 | (52) |
|  | Female | 37 | (48) |
| Age (years) | median [IQR] | 14 | [10-36.5] |
| Case | Probable | 72 | (94) |
|  | Confirmed | 5 | (6) |
| Lesion localization | Lower limbs | 36 | (47) |
|  | Upper limbs | 30 | (39) |
|  | Upper and lower limbs | 3 | (4) |
|  | Head, trunk | 8 | (10) |
| Lesion type | Active lesion | 16 | (21) |
|  | Scar with incapacity | 16 | (21) |
|  | Out of which re-educated | 13 |  |
|  | Scar without incapacity | 39 | (51) |
|  | Other | 6 | (8) |
| Associated event | Wound | 9 | (11) |
| (as reported by patient) | Insect bite | 17 | (22) |
|  | Other | 9 | (11) |
|  | Unknown | 42 | (56) |
