# Supplementary material for: Adequate Wound Care and Use of Bed Nets as Protective Factors against Buruli Ulcer: Results from a Case Control Study in Cameroon
Source: PLoS Negl Trop Dis. 2011 Nov 8;5(11):e1392. doi: 10.1371/journal.pntd.0001392 (PMC3210760; doi:10.1371/journal.pntd.0001392)
Supplement: Table S2 — Case-family matched control study analysis. Univariate and multivariate analysis results of risk factor analysis in Bankim, Cameroon, 2007–2009. (DOC) [file pntd.0001392.s003.doc]

**Table S2: Case-family matched control study analysis - Univariate and multivariate analysis results of risk factor analysis in Bankim, Cameroon, 2007-2009.**

**Table S2.1: Univariate analysis of selected demographic variables for Buruli ulcer disease in Bankim, Cameroon, 2007-2009. Family-matched case-control study.**

***Odds-Ratio, 95% confidence interval and p-value obtained using conditional logistic regression**

| **Variable** |  | **Cases** | | **Controls** | | **OR*** | **[95%CI]*** | **p*** |
| --- | --- | --- | --- | --- | --- | --- | --- | --- |
|  |  | **N** | **(%)** | **N** | **(%)** |  |  |  |
| **Gender** | Men | 21 | (47) | 24 | (53) | 1 | Reference | 0.48 |
|  | Women | 16 | (39) | 25 | (61) | 0.7 | [0.3-1.8] |  |
| **Age (years)** | median [IQR] | 11 [9-16] | | 10 [7-14] | | Matching variable | | |
| **Main activity** | School | 24 | (65) | 36 | (73) | 1 | Reference | 0.61 |
|  | Activity in the bush | 8 | (21) | 7 | (14) | 3.2 | [0.5-21] |  |
|  | Activity in the town | 3 | (8) | 3 | (6) | 2.3 | [0.3-25] |  |
|  | Child < 5 years | 2 | (5) | 3 | (6) | NA | NA |  |
| **Fishing activity** | Y/N | 21 | (59) | 22 | (57) | 0.95 | [0.3-2.7] | 0.93 |
| **Farming activity** | Y/N | 29 | (78) | 40 | (82) | 0.8 | [0.2-3.7] | 0.8 |
| **Education level** | None | 7 | (19) | 8 | (16) | 1 | Reference | 0.70 |
|  | Primary (or coranic) | 26 | (70) | 34 | (69) | 0.7 | [0.2-2.6] |  |
|  | Secondary | 4 | (11) | 7 | (14) | 0.3 | [0.04-2.9] |  |
| **BCG scar** | Present Y/N | 15 | (41) | 27 | (55) | 0.6 | [0.2-1.4] | 0.2 |

**Table S2.2: Univariate analysis of selected individual variables (insect exposure, insect protection and wound care) for Buruli ulcer disease in Bankim, Cameroon, 2007-2009. Family-matched case-control study.**

***Odds-Ratio, 95% confidence interval and p-value obtained using conditional logistic regression**

| **Variables** | | **Cases** | | **Controls** | | **OR*** | | **[95% CI]*** | **p*** |
| --- | --- | --- | --- | --- | --- | --- | --- | --- | --- |
|  |  | **N** | **(%)** | **N** | **(%)** |  |  |  |  |
| **Insect bites** |  |  |  |  |  |  | |  |  |
| **- Chrysops sp.** | Never | 3 | (8) | 7 | (14) | 1 | | Reference | 0.40 |
|  | Rare to often | 25 | (67) | 34 | (70) | 3.8 | | [0.4-35] |  |
|  | Every day | 7 | (19) | 7 | (14) | 5.2 | | [0.4-71] |  |
|  | Doesn’t know | 2 | (5) | 1 | (2) | -- | | -- |  |
| **- Simulium sp.** | Y/N | 31 | (84) | 44 | (90) | 0.6 | | [0.1-2.5] | 0.44 |
| **- cause scratch wounds** | Y/N | 26 | (70) | 28 | (57) | 2.3 | | [0.8-6.7] | 0.11 |
| **Protection using :** |  |  |  |  |  |  | |  |  |
| **- Mosquito coils (syst.)** | Y/N | 3 | (8) | 4 | (8) | 2 | | [0.2-25] | 0.55 |
| **- Bed net (syst.)** | Y/N | 9 | (24) | 18 | (37) | 0.4 | | [0.1-1.4] | 0.13 |
|  | |  |  |  |  | |  |  |  |
| **Wound treatment habits** | |  |  |  |  | |  |  |  |
| **- use leaves** | Yes/No | 19 | (51) | 16 | (33) | | 2.7 | [0.8-8.5] | 0.08 |
| **- use soap** | Yes/No | 33 | (89) | 36 | (73) | | 0.6 | [0.1-2.8] | 0.54 |
| **- use alcohol** | Yes/No | 9 | (24) | 9 | (18) | | 0.8 | [0.2-3.5] | 0.81 |
| **- use ointment** | Yes/No | 16 | (43) | 26 | (53) | | 0.7 | [0.2-1.7] | 0.41 |
| **- no treatment** | Yes/No | 6 | (16) | 8 | (16) | | 1 | [0.2-4.5] | 0.99 |
| **Treatment frequency** | No treatment | 4 | (11) | 10 | (20) | | 1 | Reference | 0.28 |
|  | ≤1 time/week | 10 | (27) | 12 | (24) | | 2.7 | [0.6-12] |  |
|  | >1 time/week | 23 | (62) | 23 | (47) | | 2.9 | [0.7-12] |  |
|  | ND |  |  | 1 | (2) | | -- | -- |  |
| **Dressing frequency** | No bandage | 19 | (51) | 20 | (41) | | 1 | Reference | 0.31 |
|  | ≤1 time/week | 5 | (14) | 11 | (22) | | 0.3 | [0.1-1.5] |  |
|  | >1 time/week | 12 | (32) | 16 | (33) | | 0.7 | [0.2-2.3] |  |
|  | ND | 1 | (3) | 2 | (4) | | -- | -- |  |

**Table S2.3: Univariate analysis of selected water exposure variables for Buruli ulcer disease in Bankim, Cameroon, 2007-2009. Family-matched case-control study.**

***Odds-Ratio, 95% confidence interval and p-value obtained using conditional logistic regression**

| **Activities** | | **Cases** | | **Controls** | | **OR*** | **[95%CI]*** | **P*** |
| --- | --- | --- | --- | --- | --- | --- | --- | --- |
|  |  | **N** | **(%)** | **N** | **(%)** |  |  |  |
| **Fish** | Never | 18 | (49) | 21 | (43) | 1 | Reference | 0.54 |
|  | Rare to often | 8 | (22) | 15 | (31) | 0.5 | [0.1-1.9] |  |
|  | Every day | 11 | (30) | 13 | (27) | 0.8 | [0.2-2.7] |  |
| **Bathe in water collections** | | |  |  |  |  |  |  |
| **-for hygiene** | Never | 9 | (24) | 15 | (31) | 1 | Reference | 0.60 |
|  | Rare to often | 9 | (24) | 10 | (20) | 1.7 | [0.4-7.1] |  |
|  | Every day | 19 | (51) | 24 | (50) | 1.9 | [0.5-7] |  |
| **Bathing place** | Barrage Y/N | 4 | (10) | 7 | (14) | 0.6 | [0.1-3.5] | 0.57 |
|  | Mbam Y/N | 4 | (10) | 2 | (4) | 3.6 | [0.4-36] | 0.23 |
|  | Other river Y/N | 19 | (51) | 27 | (55) | 0.8 | [0.2-2.9] | 0.75 |
| **-for leisure** | Never | 12 | (32) | 24 | (49) | 1 | Reference | 0.26 |
|  | Rare to often | 10 | (27) | 8 | (16) | 2.5 | [0.7-8.8] |  |
|  | Every day | 15 | (41) | 17 | (35) | 2.3 | [0.7-7.7] |  |
| **Bathing place** | Other river Y/N | 17 | (46) | 17 | (35) | 2.1 | [0.6-7.5] | 0.20 |
| **Wash clothes** | Never | 2 | (5) | 6 | (12) | 0.4 | [0.1-2.4] | 0.36 |
|  | Rare to often | 32 | (87) | 41 | (84) | 1 | Reference |  |
|  | Every day | 3 | (8) | 2 | (4) | 2.9 | [0.3-29] |  |
| **Carry water for household** | Every day | 21 | (57) | 31 | (63) | 1 | Reference | 0.82 |
|  | Rare to often | 12 | (32) | 14 | (29 | 1.3 | [0.5-3.7] |  |
|  | Never | 4 | (11) | 4 | (8) | 1.4 | [0.3-7.0] |  |

**Table S2.4: Univariate analysis of farming activity variables for Buruli ulcer disease in Bankim, Cameroon, 2007-2009. Family-matched case-control study.**

+children were considered having farming activities when they accompanied their parents to the fields;

*Odds-Ratio, 95% confidence interval and p-value obtained using conditional logistic regression

|  | | **Cases** | | **Controls** | | **OR*** | **[95%CI]*** | **p*** |
| --- | --- | --- | --- | --- | --- | --- | --- | --- |
|  |  | **N** | **(%)** | **N** | **(%)** |  |  |  |
| **Farming activities+** | Y/N | 33 | (90) | 44 | (89) | 0.8 | [0.1-4.6] | 0.76 |
| **Cultures** | Corn Y/N | 33 | (90) | 44 | (89) | 0.8 | [0.1-4.6] | 0.76 |
|  | Coffee Y/N | 12 | (32) | 16 | (33) | 1.1 | [0.2-5.6] | 0.89 |
|  | Banana Y/N | 12 | (32) | 17 | (35) | 0.9 | [0.2-3.4] | 0.9 |
|  | Cassava Y/N | 21 | (57) | 32 | (65) | 0.3 | [0.1-1.5] | 0.11 |
|  | Ground-nut Y/N | 27 | (73) | 39 | (80) | 0.5 | [0.1-2.7] | 0.40 |
|  | Beans Y/N | 6 | (16) | 9 | (18) | 0.9 | [0.2-4.6] | 0.9 |
|  | Tubers Y/N | 23 | (62) | 36 | (73) | 0.3 | [0.06-1.3] | 0.08 |
|  | Pepper Y/N | 4 | (11) | 4 | (8) | 1.3 | [0.2-8.0] | 0.8 |
| **Farming area** | Barrage Y/N | 9 | (32) | 17 | (35) | 0.2 | [0.02-1.7] | 0.08 |
|  |  |  |  |  |  |  |  |  |
| **Own a “garden”** | Y/N | 16 | (43) | 17 | (35) | 2.7 | [0.5-13.5] | 0.21 |
| **Nearby compound** | Y/N | 6 | (16) | 8 | (16) | 1.6 | [0.1-18.3] | 0.69 |
| **Water garden** | Y/N | 14 | (38) | 14 | (29) | 3.2 | [0.6-16] | 0.13 |

**NB: Many variables related to fields had identical values for cases and controls since siblings usually went to the same fields (with their parents).**

**Table S2.5: Multivariable model for risk factors for Buruli ulcer disease in Bankim, Cameroon, 2007-2009. Family-matched case-control study.**

All variables with p-values<0.25 in univariate analysis were included in the multivariate analysis. These variables were: systematic use of bed net, cassava field, wash clothes in stagnant water, treats wounds with leaves, dressing frequency, works at a garden, bathes for leisure in a river, tuber field, shoes worn at the farm, bcg scar, scratch wounds after insect bites, field location near the dam lake.

No variable was left after the stepwise variable elimination procedure. Variables were eliminated in order cited above.
