# Supplementary material for: Adequate Wound Care and Use of Bed Nets as Protective Factors against Buruli Ulcer: Results from a Case Control Study in Cameroon
Source: PLoS Negl Trop Dis. 2011 Nov 8;5(11):e1392. doi: 10.1371/journal.pntd.0001392 (PMC3210760; doi:10.1371/journal.pntd.0001392)
Supplement: Text S1 — Details on multivariate analysis (multivariable conditional logistic regression) for case-community matched control analysis of risk factors for Buruli ulcer in Bankim, Cameroon, 2007–2009. (DOC) [file pntd.0001392.s004.doc]

**Text S1: Details on multivariate analysis (multivariable conditionnal logistic regression) for case-community matched control analysis of risk factors for Buruli ulcer in Bankim, Cameroon, 2007-2009.**

Results of the univariate analysis are shown in table 1 to 4. Twenty-nine variables came out with associations characterised by P values less than 0.25. When two of these variables were highly correlated, they were tested simultaneously against the dependent variable in the same conditional logistic regression model, and were kept for the building of the final model only if still significant at the 0.25 level when tested simultaneously. This led to the identification of 11 variables that were introduced simultaneously for the building of the final model. These variables were:

- Health related factors: Using soap for cleansing wounds, Frequently changing bandages

- Agriculture related factors: Growing cassava, Having a field at risk of inundation at the rainy season, Being wounded while clearing the fields, Watering a garden,

- Water related activities (other than fishing): Wearing shoes while collecting domestic water or washing clothes, Having baths for hygiene in the Mbam river,

- Insect related exposures: Systematically using a bed net, Reporting scratch wounds after insect bites,

- Habitation related factors: Presence of a water source near the habitation.

Variables were then removed starting by those with the highest P values until all variables left in the final model had P values less than 0.05. The variables were removed in the following order:

- Presence of a water source near the compound
- Wearing shoes while collecting domestic water or washing clothes
- Having a field at risk of inundation at the rainy season
- Frequently changing bandages
- Watering a garden
- Being wounded while clearing the fields

to keep only the five variables of the final model shown in Table 5.

Of note, confounding had limited impact, and only worked to decrease the magnitude of observed associations, as can be observed when comparing raw (univariate) and adjusted (multivariate) ORs: Using soap (0.1 unchanged), Scratch wounds after insect bites (2.1 to 2.7, respectively), Systematic use of a bed net (0.45 to 0.4), Growing cassava (0.4 to 0.3), and Having baths in the Mbam river (4.4 to 6.9).
